# High Calorific Values Boron Powder: Ignition and Combustion Mechanism, Surface Modification Strategies and Properties

**DOI:** 10.3390/molecules28073209

**Published:** 2023-04-04

**Authors:** Yang Liu, Yinglei Wang, Yuezhou Liu, Baodong Zhao, Weixiao Liu, Qilong Yan, Xiaolong Fu

**Affiliations:** 1 Xi’an Modern Chemistry Research Institute, Xi’an 710065, China; liuyang0827204@163.com (Y.L.);; 2 State Key Laboratory of Fluorine & Nitrogen Chemicals, Xi’an 710065, China; 3 Science and Technology on Combustion, Internal Flow and Thermo-Structure Laboratory, School of Aerospace, Northwestern Polytechnical University, Xi’an 710072, China

**Keywords:** boron powder, ignition mechanism, combustion mechanism, surface modification, coating

## Abstract

Boron powder is a kind of metal fuel with high gravimetric and volumetric calorific values, which has been widely used in military fields such as solid propellants, high-energy explosives, and pyrotechnics. However, the easily formed liquid oxide layer can adhere to the surface of boron powder and react with the hydroxyl (-OH) group of hydroxyl-terminated polybutadiene (HTPB) binder to form a gel layer that is detrimental to propellant processing and restricts the complete oxidation of boron powder. Therefore, to improve the combustion efficiency of boron powder, the ignition and combustion mechanisms of boron powder have been studied, and surface coating modification strategies have been developed by researchers worldwide, aiming to optimize the surface properties, improve the reaction activity, and promote the energy release of boron powder. In this review, recent studies on the ignition and combustion mechanisms of boron powder are discussed. Moreover, the reported boron powder coating materials are classified according to the chemical structure and reaction mechanism. Additionally, the mechanisms and characteristics of different coating materials are summarized, and the mechanism diagrams of fluoride and metal oxide are provided. Furthermore, promising directions for modification methods and the potential application prospects of boron powder are also proposed.

## 1. Introduction

**Figure 1:**
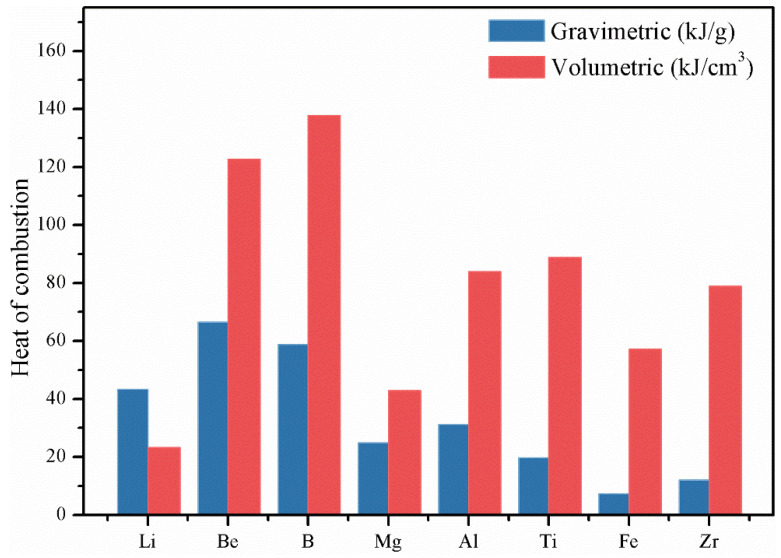
Gravimetric and volumetric calorific values of some metal fuels.

Solid propellants and explosives with much higher energy performance are urgently needed in modern national defense technology [1,2]. Metal fuels have the advantages of high energy density [3], high combustion calorific value [4], and high-frequency combustion stability [5]; therefore, they have been widely used as high-energy additives to improve the energy density of energetic formulation systems [6,7,8]. The calorific values of Be, B, Mg, Al, Li, and other high-energy metal fuels are shown in Figure 1. Among these fuels, Li and Zr are prone to spontaneous combustion and explosion in air [9]; the combustion products of Be are highly toxic [10]; and the calorific value of Fe is low. Therefore, B [11], Al [12,13,14], and Mg [15] are commonly used as metal fuels in propellants. Additionally, B is superior in calorific value to Al and Mg, as it shows a gravimetric value that is 2.1 and 1.9 times greater and a volumetric value that is 3.2 and 1.6 times greater than that of Mg and Al, respectively [16]. The calculation results show that the theoretical specific impulse of boron-containing fuel-rich propellant can reach 10,000–12,000 N·s/kg, which is 3–5 times greater than that of conventional solid propellant and about 2 times greater than that of Al–Mg fuel-rich propellant [17,18]. Therefore, boron is considered one of the most promising metal fuel additives in solid propellants [19].

In recent decades, research results have shown that three important problems have limited the application of boron [20,21], which can be summarized as follows: (1) the ignition performance is poor due to the protective oxide layer on the surface of boron powder, which thickens gradually during combustion, and resulting in a longer ignition delay time; (2) the low melting point (718 K) and high boiling point (2316 K) of B_2_O_3_ facilitate the formation of a liquid film, which attaches on the surface of boron particles during combustion, and thus hinders the diffusion of oxygen and reduces the combustion efficiency of boron (both melting point and boiling point of pure boron >2500 K) [22,23]; (3) impurities such as B_2_O_3_ and H_3_BO_3_ can react with the hydroxyl of hydroxyl-terminated polybutadiene (HTPB) and form a gel layer on the surface of boron powder, which results in poor compatibility between boron and HTPB [24,25].

Based on the aforementioned issues, boron faces challenges in unleashing its full energy release potential. To enhance the ignition performance and combustion efficiency of boron powder, a significant amount of research has been conducted [26,27,28,29,30,31]. In early studies, research efforts primarily focused on investigating the ignition and combustion mechanisms, given the intricacy of boron’s ignition mechanism relative to other metals. The challenge lies in the diffusion and reaction mechanism of B and O_2_ in the presence of an oxide layer. Four different ignition mechanisms have been proposed; however, a unified ignition mechanism has yet to be established. In recent times, there has been a growing research interest in the preparation of coated or modified boron powder. This methodology is designed to enhance the performance of boron powder by leveraging the interaction between boron powder and the coating agents or modifiers. For instance, the presence of fluorine can facilitate the rapid removal of the oxide layer that forms on the surface of boron, while certain energetic materials can generate substantial heat to accelerate the ignition of boron.

In this review, the recent development of surface modifications and the ignition and combustion mechanism of high-energy boron fuel is discussed in detail. Firstly, the ignition and combustion mechanism of boron is reviewed and summarized. Secondly, the coating materials of boron powder are divided into energetic materials, fluoropolymers and inorganic fluorides, and metals and metal oxides. Additionally, the research progress of these coating materials is reviewed. Thirdly, the performance and characteristics of different coating materials are analyzed and discussed. Promising directions of boron particle coating modification methods are also proposed, which might provide valuable references and useful suggestions for researchers in the propellant and explosive field.

## 2. Ignition and Combustion Mechanism of Boron Particles

The ignition and combustion mechanism of boron particles was initially established by Macek and Semple [32], and the mechanism indicated that the combustion of boron particles always exhibits two consecutive stages. In the first stage (or ignition stage), the boron particles are heated to about 1900 °C, showing a brief brightening and then darkening, while the boron remains covered by a pre-existing liquid B_2_O_3_ layer. As the temperature continues to increase, the second stage (or combustion stage) begins, and the boron particles start to glow again. The glow is brighter, and the boron particles show stronger reactivity than in the first stage. The combustion in the second stage is ascribed to the full combustion of the bare boron particles. The mechanism of boron ignition and combustion can be summarized and analyzed as follows.

### 2.1. Ignition Mechanism of Boron Particle

#### 2.1.1. King’s Model

In order to explore the ignition and combustion characteristics of boron particles, Macek and Semple [32,33,34] carried out combustion experiments on crystalline boron with a diameter of 34.5–124 μm, using a flat-flame burner and a CO_2_ laser igniter. The experimental results showed that the burning rates of samples are correlated to the diffusion rate of gaseous oxidants from the surface of samples, and the burning time decreases with the increment in ambient gas temperature and oxygen mole fraction, which is consistent with the theory of gas-phase diffusion. In addition, shock-tube technology was used by Uda [35] in the ignition of crystalline boron particles with a diameter of 30–50 μm. The study shows that the ignition temperature is strongly correlated to the air pressure, and the ignition temperature increases with the air pressure drop.

To explain the above experimental phenomena, King [36,37,38] proposed the King ignition model (Table 1), which is based on the diffusion mechanism, where oxygen diffuses through liquid boron oxide to the B (s)-B_2_O_3_ (l) interface and reacts with boron. In this diffusion mechanism, Mohan and Williams [39] proposed a planar model to illustrate the ignition stage of boron; the limiting oxygen mass fraction predicted by this model is very close to the result observed by Prentice [40]. The critical ignition temperature of boron particles calculated by the ignition stage model proposed by Meinkohn [41] and based on the King model is 1900 K, which is in good agreement with the experimental results of Macek [32]. Based on King’s model, Gaponenko and Meese also proposed their own models and achieved good prediction accuracy [42,43].

#### 2.1.2. L-W Model

To further understand the ignition mechanism of boron particles, Glassman [44] calculated and compared the solubility and the diffusion rate of B (s) and O_2_ (g) in B_2_O_3_ (l) using a solubility model. The results showed that the solubility of O_2_ (g) in B_2_O_3_ (l) is 10 orders of magnitude smaller than that of B (s) in the temperature range of 1800–2300 K. Therefore, the diffusion mechanism proposed by Glassman suggests that boron is dissolved in liquid boron oxide and forms a compound similar to B_2_O_X_ (X < 3) at the ignition stage, followed by diffusion to the boron oxide–gas interface to react with oxygen. On the basis of diffusion mechanisms, Li and Williams [45,46] further proposed an L-W model (Table 1). This model involves four global reactions during the ignition stage: (1) the formation of BO (d) by the dissolution equilibrium of B in the B_2_O_3_ (l) layer; (2) the reaction of O_2_ (g) and H_2_O (g) with BO (d) to form BO_2_ (g) and HOBO (g); (3) the vaporization of B_2_O_3_ (l); and (4) the reaction of O_2_ with bare B after removal of the oxide layer to form O=B–B=O bonds. The prediction of the above model is consistent with the experimental results of Macek’s research on flat-flame burners and laser ignition tests [33,34]. Notably, the energy released during the combustion of boron particles undergoes a significant reduction due to the emergence of HOBO (g). This is because only when all the final products are converted into thermodynamically favorable B_2_O_3_ (l) will the energy of boron be fully released. However, the sluggish conversion of HOBO (g) to B_2_O_3_ (l) decelerates the energy release rate in the presence of oxygen and hydrogen.

#### 2.1.3. Y-K Model

In 1996, Yeh and Kuo [47] summarized and established a Y-K model based on environmental scanning electron microscope (ESEM) and X-ray diffraction (XRD) analysis of reaction products during the ignition and combustion processes of small boron particles. The experimental results showed that the diffusion of B in B_2_O_3_ (l) was dominant at higher temperatures, the rationality of which was verified by Glassman and Li. Unlike the L–W model, the Y-K model suggested that the main evaporation product is B_2_O_2_ rather than B_2_O_3_, and the chemical reaction mechanism involved is shown in Table 1. The ignition delay time of boron particles with different particle sizes (d = 3 μm and d = 34.5 μm) calculated by Y–K model agrees well with the experimental results. For other sizes of boron particles, based on the Y-K model, Ermolaev [48] studied the combustion of large particles (d = 34.5 μm and d = 44.2 μm) of boron in hot gas and improved the error accuracy to 20%. Bedarev [49] simulated the ignition and combustion of boron particles with a diameter of 7.5~20 μm after a shockwave reflected from the wall. The model considers the boiling process of boron. The ignition and combustion kinetics of the particles were obtained, including the heating and melting of the boron trioxide oxide layer on the particle surface; the evaporation and heating of pure boron particles; and the melting, boiling, and the corresponding chemical reactions. The model is qualitatively consistent with the experimental data at temperatures from 2200 to 3000 K.

Additionally, Ulas and Kuo [50] supplemented the Y-K model in 2001, and the model was extended to fluorine-containing environments because F has a significant promoting effect on the ignition and combustion of B [51]. In this case, in addition to the reaction in Table 1, the following two reactions will occur:

B (s) + B_2_O_3_ (l) + HF + O_2_ (g) → OBF (g) + FBOH (g) + HBO_2_ (g)

B (s) + B_2_O_3_ (l) + 3F (g) → 3OBF (g)

Moreover, Chen [52] established a more perfect extended ignition model in 2017, which further considered the dynamic balance between the consumption of (BO)_n_ on the oxide layer surface and the internal diffusion process during the ignition stage. In this model, the consumption–competition mechanism of (BO)_n_ was introduced after analyzing the heterogeneous reaction and evaporation process on the surface. Additionally, the influence of forced convection on particles was also considered.

#### 2.1.4. B-D Model

In 2014, Aowen [53] confirmed for the first time that oxygen can also diffuse through B_2_O_3_ based on the TEM characterization, suggesting that both (BO)_n_ and oxygen diffusion exist in the ignition process. Subsequently, they proposed a B-D (bi-direction) model for boron ignition, which involves four kinetic processes: (1) the evaporation of B_2_O_3_ (l); (2) the surface reaction of O_2_ (g) with boron in the environment; (3) the reaction of internal core boron with O_2_ (g); and (4) the overall reaction of boron with H_2_O (g). Additionally, the B-D model can be divided into two different submodels depending on the thickness of the oxide layer. In the case of a thin oxide layer, submodel Ⅰ was proposed. In this model, the particle exhibits a four-layer structure of B-B_2_O_3_-(BO)_n_-B_2_O_3_ from inside to outside, and two-way diffusion is involved. On the other hand, submodel Ⅱ is available in cases of a thicker oxide layer. In this model, the particle shows a three-layer structure of B-(BO)_n_-B_2_O_3_ from inside to outside, and (BO)_n_ adopts a unidirectional diffusion behavior.

In addition, Dreizin et al. [54,55] proposed another boron ignition model. The model supposes that oxygen dissolves in solid boron and gradually reaches its solubility limit in the ignition stage of boron particles. Once the solubility limit is reached, the boron particles will enter the combustion stage and exhibit strong heat release. However, there were no B–O phase diagram data to provide sufficient evidence for this model until now [56].

**Table 1:** Four ignition models of boron particles.

Models	Reaction Mechanism of Boron Particles at the Ignition Stage
King’s model [36,37,38]	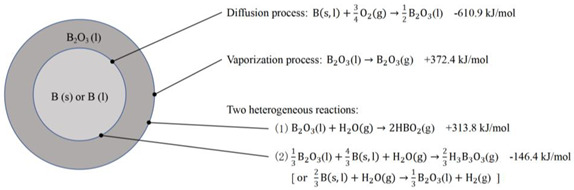
L-W model [45,46]	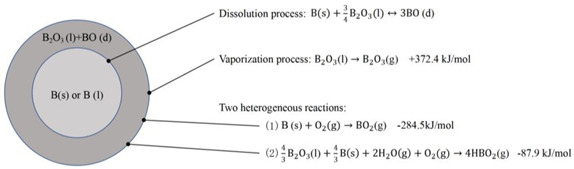
Y-K model [47]	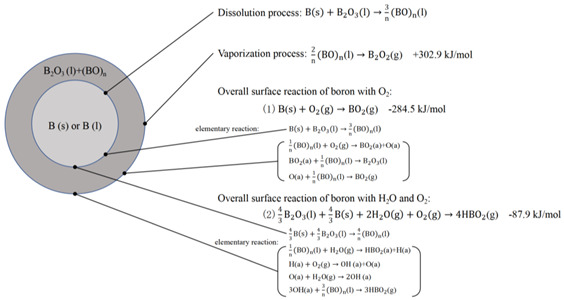
B-D model [53]	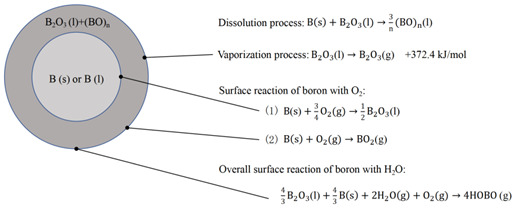

### 2.2. Combustion Mechanism of Boron Particles

In general, the combustion stage of boron particles is simpler than the ignition stage, which adopts a predominant heterogeneous surface reaction [57]. On the basis of experimental data of combustion studies on large boron particles (d > 35 μm), Macek and Mohan [32,39] believed that the combustion of large boron particles was controlled by diffusion and conformed to the d^2^ law. In other words, the combustion time of large boron particles was proportional to the square of particle size, which was similar to that of hydrocarbon droplets.

King [58] thought it impossible for the diffusion transport rate to approach infinity with the decrease in particle size only through diffusion control during boron combustion. Based on King’s theoretical research and data analysis, it was found that the combustion stages of large and small boron particles were controlled by diffusion (d^2^ law) and chemical kinetics (d^1^ law), respectively. King roughly estimated that the transition from d^2^ law to d^1^ law was approximately 15–30 μm in diameter of boron, and the exact transition point depended on pressure.

Li and Williams [45,59] also observed that the combustion of small boron particles (7 and 10 μm) was controlled by chemical kinetics (d^1^ law). In addition, it was found that the combustion time was equal to the sum of the time that was needed for oxygen diffusion to the surface of boron particles and for boron surface oxidation (2B + O_2_ → B_2_O_2_). Based on this discovery, the L-W combustion model considering both the gas diffusion process and chemical kinetics was proposed.

**Figure 2:**
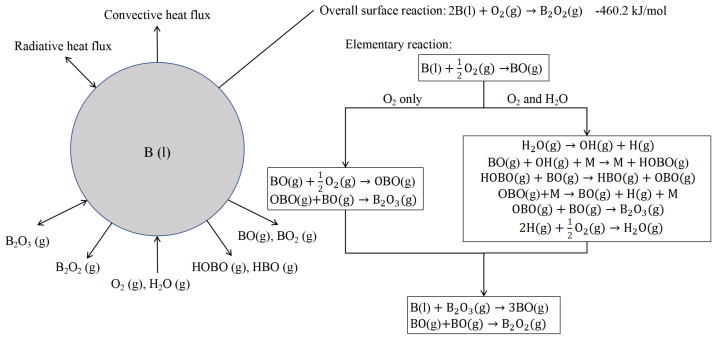
Reaction mechanism of boron particle combustion in the Y-K model [47].

To determine the critical conditions of diffusion control and kinetic control, the Y-L combustion model was improved by Kuo [47,50], as shown in Figure 2. The concept of the Damkohler number (Da) is proposed to determine the control mechanism of the boron combustion stage. The Da, which is widely used in chemical engineering to characterize chemical reaction kinetics in various systems, is usually defined as the ratio of the characteristic time of a chemical reaction to the characteristic time of diffusion of reactants or products. In mathematics, this can be expressed as τt/τr, where τr is the characteristic time of the chemical reaction and τt is the characteristic time of diffusion. For the combustion reaction of boron particles, the Da number can be expressed as Equation (1):

(1) $$\mathrm{Da}=\frac{t_{dif}}{t_{kin}}$$

where *t_dif_* is the combustion time under diffusion control and *t_kin_* is the combustion time under kinetic control. 

It is assumed that the initial diameter of boron particles is d_0_ and the density is ρ_B_. For the case of diffusion control, the ignition of boron particles is controlled by the diffusion of oxygen in the environment. It can easily be determined that the volume consumption rate, V_dif_, of boron particles per unit area is shown in Equation (2), which is the same as the change in radius relative to time. Therefore, the differential equation can be obtained. The combustion time *t_dif_* can thus be determined as Equation (4):

(2) $$V_{\mathrm{dif}}=\frac{m}{4\pi \rho_{B}r^{2}}=\frac{\rho Dln\left(1+iY\right)}{r\rho_{B}}$$

(3) $$\frac{dr}{dt}=V_{\mathrm{dif}}$$

(4) $$t_{dif}=\frac{\rho_{B}d_{0}^{2}}{8\rho Dln\left(1+iY\right)}$$

where *m* is the mass of boron particles, *r* is the radius of boron particles, *ρD* is the product of gas density and diffusion, *i* is the mass stoichiometric ratio of fuel and oxidant, and *Y* denotes the mass fraction of oxygen in the ambient.

In another kinetically controlled combustion mechanism, it can be assumed that the diffusion rate of O_2_ is much faster than the reaction rate on the particle surface. At this time, the volume consumption rate, V_kin_, of boron particles per unit area can be written as Equation (5), and the combustion time, *t_kin_*, under kinetic control can be obtained by an integral using the same method as Equation (6):

(5) $$V_{\mathrm{kin}}=M_{B}kPX$$

(6) $$t_{kin}=\frac{\rho_{B}d_{0}}{2M_{B}kPX}$$

where *X* is the mole fraction of oxygen, *k* is the reaction rate constant, *P* is the environmental pressure, and *M_B_* is the relative atomic mass of boron. Since *iY* ≪ 1, ln (1 + *iY*) is approximately equal to *iY*. Substituting the data into the Da expression yields Equation (7) [47]:

(7) $$\mathrm{Da}=\frac{t_{dif}}{t_{kin}}=\frac{Pd_{0}M_{B}kX}{4\rho DiY}=\frac{Pd_{0}}{75}$$

The kinetic controlled combustion time equals the diffusion-controlled combustion time when Da = 1 or *pd*_0_ = 75 atm-μm. In this case, the combustion of boron particles is controlled by both oxygen diffusion and kinetics. When Da ≪ 1.0 or *pd*_0_ ≪ 75 atm-μm, the combustion of boron particles is mainly controlled by kinetics. When Da ≫ 1.0 or *pd*_0_ ≫ 75 atm-μm, the combustion of boron particles is mainly controlled by the oxygen diffusion process. This suggests that the combustion stage is dominated by diffusion control under high pressure and large particles, while it is dominated by kinetics control under low pressure and small particles. In order to enrich the application range of the Y-K combustion model, Kuo [50] extended the model to the occasion that O_2_, H_2_O, F, HF, and other gases exist. The extended model is highly consistent with the experimental data and has been widely used.

Hussmann and Pfitzner [60,61] further considered the influence of forced convection and boron evaporation in the combustion process, and made up for the shortcomings and defects of the Y-K model. Therefore, a transient model of a ramjet combustion chamber was established, which can be used for the calculation of three-dimensional computational fluid dynamics (CFD).

## 3. Surface Modification of Boron Powder

**Figure 3:**
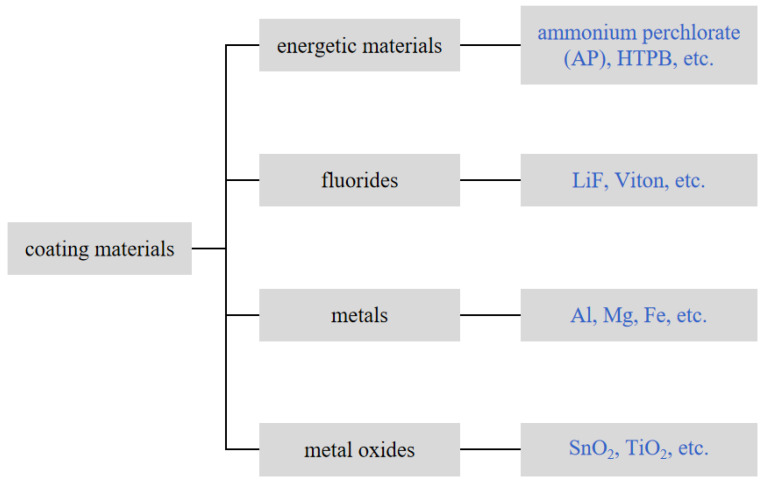
Classification of commonly used materials for surface coating modification of boron powder.

In addition to studying the ignition and combustion mechanism of boron particles, surface modification strategies are beneficial to improving the combustion efficiency of boron powder. Surface modifications of boron powder can improve the surface properties and increase the energy and reactivity of energetic systems. The commonly used coating materials are shown in Figure 3.

### 3.1. Boron Powder Surface Modification with Energetic Material

Energetic materials can release a considerable amount of heat during combustion, which can improve the surface temperature and the ignition and combustion properties of boron [62,63,64,65]. The energetic materials used for boron powder coatings are mainly divided into oxidizers and binders.

#### 3.1.1. Boron Powder Coated with Oxidizer

##### Boron Coated with Ammonium Perchlorate (AP)

**Figure 4:**
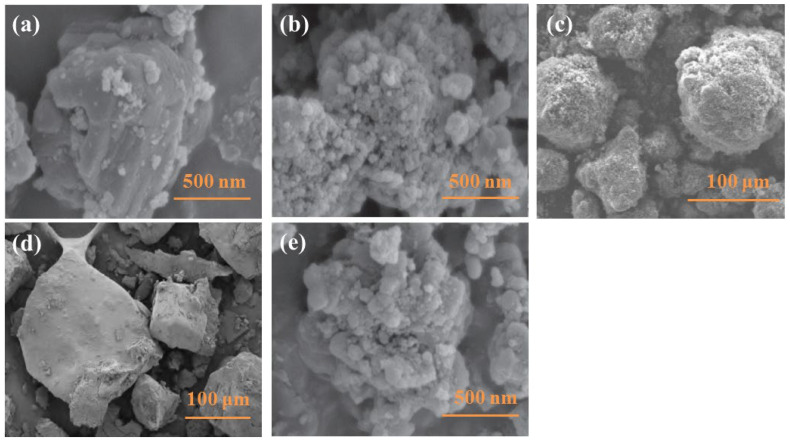
SEM images of (**a**) raw boron [71]; (**b**) B@AP-10% [71]; (**c**) B@AP-50% [72]; (**d**) B@AP/LiP-36%/44% [73]; and (**e**) B@NQ-10% [71].

AP is a common component in solid propellant [66,67], which has the advantages of high formation enthalpy, excellent stability, and being a cheap and abundant resource of raw materials [68,69]. Therefore, many studies on AP-coated boron powder have been carried out. Zhang et al. [70] investigated the effect of surfactant and preparation processes on the properties of AP-coated boron powder. The results indicated that at an evaporation rate of 10 g·h^−1^, the use of organic solvent and a silane coupling agent before coating will lead to the more uniform deposition of coating materials on the surface of boron particles. As Figure 4 shows, the morphology of boron particles changes significantly after coating. The raw boron particles without AP coating show a relatively smooth surface and an irregular shape (Figure 4a) [71]. However, B@AP particles coated with 10 and 50% of AP show a surface that is full of small coating agent grains (in Figure 4b,c). The AP coating not only increases the specific surface area of boron particles, but also acts as an oxidant by replacing oxygen to react with the boron core [71,72]. Different from AP-coated surfaces, the surface of AP/lithium perchlorate (LiP) co-coated B is tight and smooth (Figure 4d), and no small grains are found [73]. The compatibility of boron powder can be improved by AP coating in HTPB systems due to changes in surface characteristics [74].

**Figure 5:**
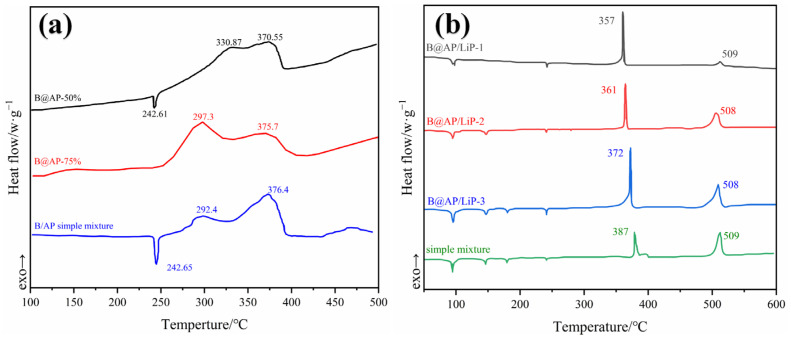
The DSC curves of experimental samples (**a**) in an air atmosphere [29,75]; and (**b**) in a N_2_ atmosphere (the crystallization temperatures of B@AP/LiP-1, B@AP/LiP-2, and B@AP/LiP-3 are 60 °C, 70 °C, and 80 °C, respectively [73]).

Wang et al. [29] and Hu et al. [75] investigated the thermal reaction performance of B@AP particles. According to the DSC curves of B@AP-50% and B@AP-75% in Figure 5a, the peak temperature, peak area, and heat release at the low-temperature decomposition stage of the B/AP mixture sample (1415 J/g) were decreased compared with those of the pure AP sample (1835 J/g). This can be attributed to the covering of the low-temperature decomposition peak caused by heat released from the reaction between boron and the decomposition products of AP. On the other hand, the peak high-temperature decomposition stage of B/AP is higher than that of pure AP (352.8 °C [29]), which can be attributed to the increased diffusion resistance and the reaction lags induced by boron oxide film formation. However, after optimized coating modification, the diffusion resistance can be decreased, and the peak temperature of B@AP-50% and B@AP-75% is lower than the optimized results, suggesting that boron in B@AP particles can promote the decomposition reaction of AP, thus increasing the heat released by B@AP-coated particles during the thermal decomposition process.

AP + LiP-co-coated B was prepared by Gao [73] to solve the performance defects of a single oxidant (lower oxygen supply of AP and easy moisture absorption of LiP). The AP decomposition exothermic peak temperatures of the coated particles with different crystallization temperatures are advanced in a certain range (up to 30 °C) relative to the simple mixture samples (Figure 5b). The results further indicate that the coating modification has a catalytic effect on the decomposition of AP.

##### Boron Coated with Octogen (HMX) and Nitroguanidine (NQ)

**Figure 6:**
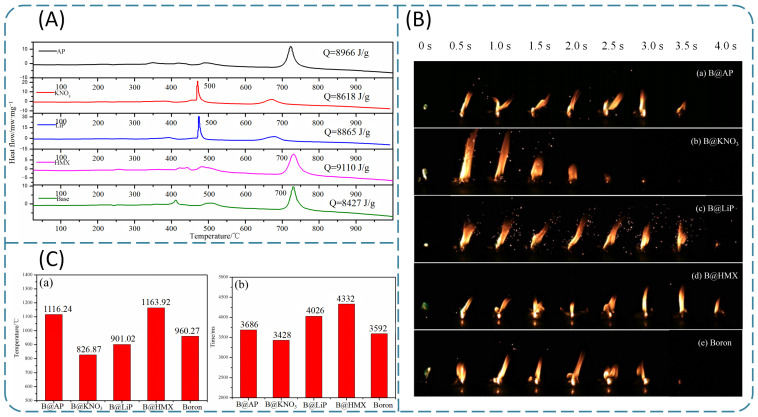
(**A**) The DSC curves and (**B**) combustion flame images of different samples; (**C**) combustion characteristics of (**a**) average combustion temperature and (**b**) average self-sustaining combustion time in the laser ignition experiment [76].

HMX, NQ, and other high-energy explosives have been used as coating materials to improve the energy of components due to their excellent detonation performance and higher energy density compared with oxidizers. Liang et al. [76] prepared four types of coated boron powder using HMX, AP, LiP, and KNO_3_ as coating materials. As shown in Figure 6A, KNO_3_ and LiP coatings effectively reduced the initial reaction temperature of the samples. In the whole thermal oxidation process, the order of total heat release values of coating materials and raw boron is HMX > AP > LiP > KNO_3_ > raw boron. In addition, flame morphologies, such as flame color, flame shape, and flame size, are shown in Figure 6B. A green flame was caused by the combustion of boron (an intermediate product, BO_2_). Additionally, the flame shape of raw boron was unstable, similar to that of B@AP. Moreover, the flame size reflects the combustion intensity, and these images suggest that B@KNO_3_, B@LiP, and B@HMX have high combustion intensity, while the combustion time of B@KNO_3_ is shorter. Additionally, it can be observed from Figure 6C that the average combustion temperature, which refers to the average value of the measured temperature data during the entire combustion process, and the combustion time of HMX-coated samples, exhibited the highest values of 1163.92 °C and 4.3 s, respectively. Overall, the HMX coating can improve the combustion performance of boron samples most effectively.

**Figure 7:**
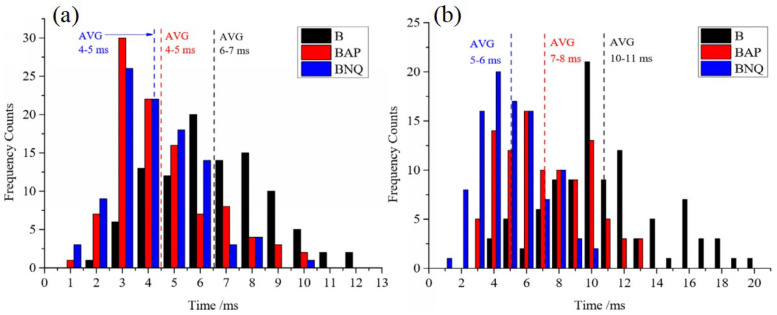
(**a**) Ignition time frequency counts and (**b**) combustion time frequency counts of different samples at 10 atm [71].

In order to further improve the reaction activity of boron powder, Liang et al. [71] prepared B@AP and B@NQ by the recrystallization method. Figure 4e shows that many irregular coating agent grains were deposited on the surface of B@NQ. The burning characteristics of the sample were also measured using the jet flow pressurized concentrated ignition experimental system. As shown in Figure 7, the average ignition time and combustion time of B@NQ decreased by 35 and 53% compared with raw boron, respectively. Faster burning represents a more intense reaction; thus, it can be deduced that the NQ coating is better than the AP coating. Therefore, the relatively higher energy density of HMX and NQ can improve the combustion performance of boron powder more preferably.

#### 3.1.2. Boron Powder Coating with Binder

3,3-bis (azidomethyl) oxetane/tetrahydrofuran copolymer (PBT) and glycidyl azide polymer (GAP) are two typical azide energetic binders [77,78]. Azide binders have several advantages, such as high heat release, no oxygen consumption during decomposition, and low molecular weight of decomposition products [79]. The direct use of boron powder in solid propellant can result in a gelation reaction that severely affects the charging process of the propellant (i.e., the preparation process of solid propellant). Viscosity is a crucial parameter that characterizes this process, and the binder, as the main component of solid propellant, typically has a low viscosity to facilitate charging. Therefore, the principal advantage of boron powder coated with a binder is that it improves the charging process of solid boron-based propellants and enhances the compatibility of B with HTPB.

##### Boron Coated with PBT

Zhang [80] studied the effect of B@PBT on the charging process of boron-based propellants. The results of the acidity analysis showed that the pH values of raw boron and B@PBT were 3–4 and above 6.50, respectively, which indicated an obvious acidity decrease in B@PBT. Table 2 shows that the viscosity of B@PBT/HTPB is much lower than that of B/HTPB. With the improvement in charge process performance, the combustion efficiency of the propellant is also improved. Less unburned boron powder was detected in the propellant combustion residues.

##### Boron Coated with GAP

**Figure 8:**
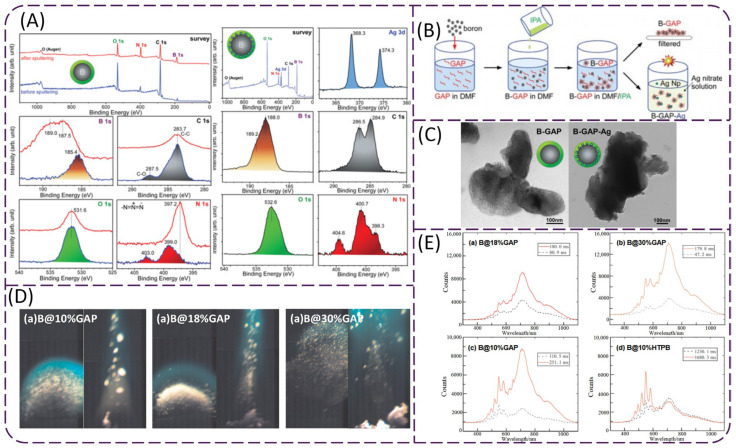
(**A**) Survey and high-resolution (B 1s, C 1s, O 1s, and N 1s) XPS spectra of B@GAP and B@GAP-Ag [82]; (**B**) schematic diagram of the coating procedures [82]; (**C**) TEM images of B@GAP and B@GAP-Ag [82]; (**D**) the ignition and combustion process of B@GAP [84]; (**E**) spectra of B@GAP and B@HTPB during the ignition and combustion processes [84].

The solid fuel-rich propellant based on B@GAP, which was prepared using the solvent–nonsolvent method, also shows the advantage of reduced slurry viscosity during propellant blending and casting [81]. Moreover, the ignition/combustion time of B@GAP powder was also measured, revealing that the ignition delay times for raw boron and coated B@GAP were 16.2 ms and 7.6 ms, respectively. In another study, Shin [82] prepared core–shell-structured B@GAP particles through the solvent–nonsolvent method using N, N-Dimethylformamide (DMF) as the solvent and isopropanol (IPA) as the nonsolvent. Furthermore, B@GAP was dispersed in silver nitrate solution to prepare silver-modified B@GAP-Ag particles under ultraviolet irradiation for 1 hour. The preparation process and the microstructure of coated particles are presented in Figure 8B,C. The XPS results in Figure 8A show that the B1s peak value (185.4 eV) of B@GAP is lower than that of bare boron (187.9 eV). This is because the active azide (-N_3_), an electron donor group in GAP, is located near the surface of electron-deficient boron, and can thus form the B-N_3_-R bond with boron. The authors believe that the position of N_3_ may further enhance the combustion ability of boron. The spectrum of B@GAP-Ag shows a new N 1s peak at 398.3 compared with that of the B@GAP, indicating the formation of a N = C bond on the GAP surface after GAP had been irradiated by light [83].

Deng [84] studied the combustion characteristics of B@GAP. It can be seen from Figure 8D,E that GAP can improve the ignition and combustion performance of boron powder. As the GAP content changed from 10 to 30%, the flame volume of B@GAP increased and the combustion became more intense. The combustion spectral intensity of B@GAP also increased, and the ignition delay time decreased from 100.7 to 45.1 ms. In addition, at a coating agent content of 10%, the average ignition delay time of B@GAP was 100.7 ms, which is much shorter than that of B@ HTPB (1300 ms). Moreover, Fan [85] studied a boron-based fuel-rich propellant containing B@GAP. The results showed that B@GAP reduced the content of aluminum and AP in the combustion residues of the propellant, and the dispersion of the combustion residues was also improved. B@GAP can also improve the combustion efficiency and the injection efficiency of boron-based propellant. Therefore, using GAP to coat boron powder is also a potential method to improve the energy release efficiency of boron-containing propellant.

### 3.2. Boron Powder Surface Modification with Fluoride

Fluorine is the most electronegative element in the periodic table of elements; thus, it can create a stronger oxidation environment than oxygen [86]. The fluoride gravimetric and volumetric combustion heat values of boron are 105.01 kJ/g and 245.72 kJ/cm^3^, respectively [87], which are almost doubled when compared with the oxidation heat. The most commonly used inorganic fluorides and fluoropolymers include LiF, BiF_3_, fluorographene (FG), and PVDF.

#### 3.2.1. Boron Powder Coated with Inorganic Fluoride

##### Boron Coated with LiF and BiF_3_

The coating of metal fluorides usually adopts the neutral precipitation method. Using this method, Zhang [88] prepared a uniform LiF deposition layer on the surface of boron powder using LiOH and HF as precursors. B@LiF improved the charging process of HTPB propellant. The pH of the B@LiF suspension system was increased from 4 to 7.4, and the viscosity of the HTPB system was considerably reduced. LiF can also accelerate the removal rate of B_2_O_3_ and improve the combustion efficiency [89]. The ignition delay time decreased from 70.475 to 23.585 s before and after coating in boron-containing propellant by electric wire heating ignition.

**Figure 9:**
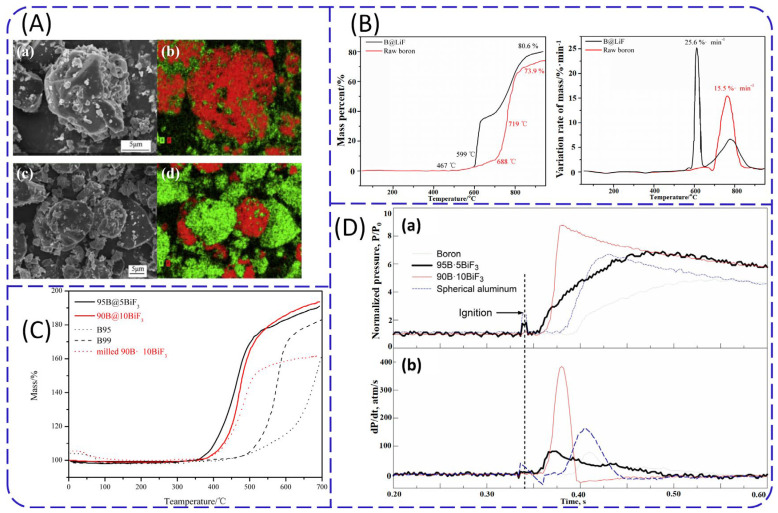
(**A**) SEM and elemental mapping images of (**a**) and (**b**) B@LiF; (**c**) and (**d**) B/LiF [90]; (**B**) TG-DTG curves of boron and B@LiF [91]; (**C**) TG curves of B@LiF, B, and milled B-BiF_3_ [92]; (**D**) constant-volume explosion experiments of B, 90B@10BiF_3_, 95B@5BiF_3_, and spherical aluminum powder (**a**) normalized pressure and (**b**) pressure change rate curves [92].

The morphology of B@LiF prepared using LiOH and NH_4_F as precursors is shown in Figure 9A [90]. It can be seen that the distribution of elemental F on the surface of coated samples is more uniform than that of mechanical mixed samples. The thermogravimetry–derivative thermogravimetry (TG-DTG) curves displayed in Figure 9B showed that the initial reaction temperature of B@LiF advanced from 719 to 599 °C, respectively, and the total weight gain increased from 73.9 to 80.6%, respectively [91]. In general, LiF coating effectively improved the thermal reaction performance of boron. According to the thermodynamic calculation [91], LiF promotes the thermal oxidation of boron powder above 1353 °C by consuming the surface oxide layer through the following reaction: B_2_O_3_ (l) + LiF (l) → LiBO_2_ (l) + BOF (l). In this situation, F^−^ in LiF can coordinate with B by replacing O^2−^ in B_2_O_3_, and disrupt the dense structure of the three-dimensional B_2_O_3_ network. As a result, the viscosity of liquid B_2_O_3_ and the diffusion resistance of O_2_ were reduced, and the thermal oxidation of boron powder was promoted. However, at temperatures lower than 1353 °C, the above reaction cannot occur spontaneously.

BiF_3_ can also be used for coating boron powder with a neutral precipitation method. By directly coating the BiF_3_ generated in situ onto the boron powder surface, Valluri et al. [92] prepared 90B@10BiF_3_- and 95B@5BiF_3_-coated particles with mass ratios of 90:10 and 95:5, respectively. The thermal analysis in Figure 9C suggests that the TG curves of B@BiF_3_ were similar to that of raw boron, but the initial reaction temperatures of 90B@10BiF_3_ and 95B@5BiF_3_ were 100 and 200 °C lower than those of raw boron, respectively. As shown in Figure 9D, in the constant-volume explosion experiments, the ignition delay time for both coated samples (0.36 s) decreased by 12% when compared with pure boron (0.41 s), and the increase in peak pressure and pressure rise rate of the coated particles were obviously higher than pure boron. The strongest pressure pulse was observed in 90B@10BiF_3_-coated particles, and the peak pressure was approximately 9 atm. The peak pressure of 95B@5BiF_3_-coated particles and spherical aluminum powder was approximately 6.5 atm, and the peak pressure of pure boron powder was the lowest.

**Figure 10:**
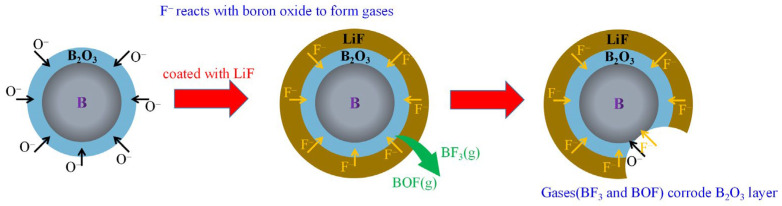
The reaction mechanism of B@LiF.

Previous studies illustrated that fluoride can enhance the reactivity of boron powder, and the interaction mechanism between fluorine and boron can be depicted in Figure 10 (using LiF as an example). Below the boiling point of boron, boron and oxidant migrate outside or inside through the B_2_O_3_ (l) layer. Due to the strong diffusion resistance, the exothermic reaction on boron powder surfaces is slow. After LiF coating, LiF produces F^−^ in the combustion process. F^−^ replaces O^−^ and reacts with B and B_2_O_3_ to produce gases such as BF_3_ and BOF, which can greatly improve the gasification and removal rate of the oxide layer. The diffusion resistance is greatly reduced, and the exposed boron in the inner layer can directly react with the oxidant, promoting the ignition and combustion of boron.

##### Boron Coated with Fluorographene (FG)

FG is an exciting novel material. The introduction of F atoms on graphene endows fluorographene with outstanding thermal stability (decomposition temperature between 400 and 600 °C [93]) and a high fluorine content (−60 wt.%) [94], which can lead to high reactivity between boron and FG.

**Figure 11:**
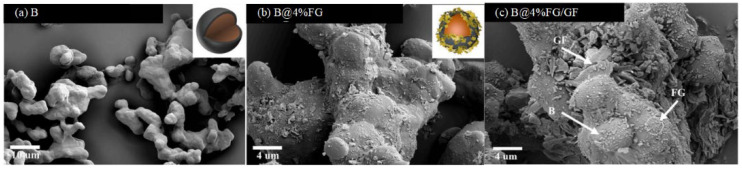
SEM images of (**a**) raw boron; (**b**) B@4%FG; and (**c**) B@4%FG/GF [95].

**Figure 12:**
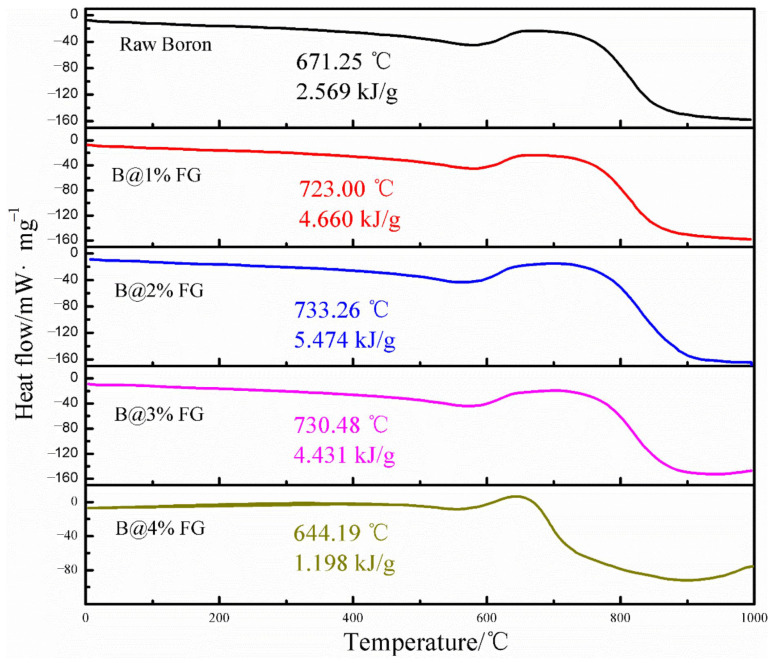
DSC curves of raw boron and B@FG/GF [95].

It can be seen from Figure 11 that FG has a considerable influence on the morphologies of boron [95]. Irregular boron particles have a relatively smooth surface (Figure 11a). In comparison, the surface of B@FG is relatively rough (Figure 11b). After adding graphite fluoride (GF) as an oxidant, the lamellar structure of GF can be observed from scanning electron microscopy (SEM) images (Figure 11c) of B@FG/GF. Additionally, GF is uniformly distributed on the surface of boron particles. In early research [96], GF showed advantages in enhancing the combustion performance of boron powder. Additionally, B@GF exhibited excellent thermal properties. The DSC curves of B@FG/GF are shown in Figure 12. As the content of FG increased from 0 to 4 wt.%, the heat release of B@FG increased first and then decreased. The maximum heat release was obtained by B@2%FG (5.474 kJ/g), which was higher than that of B/GF (2.569 kJ/g). The combustion performance was further studied, and the ignition delay time of B@GF/GF was reduced to 262 ms compared with B/GF (380 ms).

#### 3.2.2. Boron Powder Coated with Fluoropolymer

**Figure 13:**
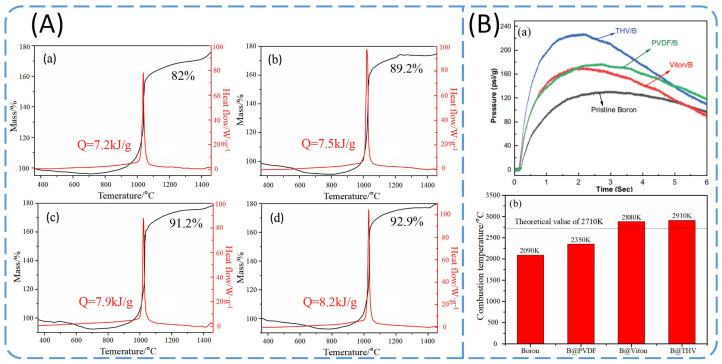
(**A**) TG and DSC curves of (**a**) raw boron; (**b**) B@PVDF; (**c**) B@Viton, and (**d**) B@THV; (**B**) (**a**) pressure curves of closed burner and (**b**) combustion temperatures in air for raw boron and fluoropolymer-coated boron powder [97].

Compared with metal fluorides, fluoropolymers have a higher process ability and operational safety, and are more easily accepted as formula additives. Keerthi [97] prepared boron powder coated with polyvinylidene difluoride (PVDF), Viton, and fluoroplastic (THV) using the solvent evaporation method; the order of fluorine content was PVDF (59 wt.%) < Viton (66 wt.%) < THV (72 wt.%). The thermogravimetry–differential scanning calorimetry (TG-DSC) curves and combustion characteristics of raw boron and three types of coated boron powder are shown in Figure 13. The results suggest that a higher fluorine content of the fluoropolymer would contribute to the better oxidation and combustion performance of boron powder. B@THV had the highest fluorine content; therefore, it showed the greatest improvement in properties such as oxidation heat (8.2 kJ/g), combustion reactivity, and combustion temperature (2910 K), followed by B@Viton and B@PVDF. However, all these coated boron powder materials demonstrated better performance than raw boron.

THV generates fluorine-rich species during thermal decomposition in an oxygen environment, and the produced gases can react strongly with the boron oxide shell to form OBF (g) in addition to high energy release and high temperature. Therefore, the oxidation and combustion performance of B@THV are greatly improved. However, PVDF has a higher hydrogen content and produces more HF (g) during thermal decomposition, and the produced HF has poor reactivity with the boron oxide shell, thus leading to only a slight improvement in the oxidation and combustion performance of B@PVDF. Additionally, B@Viton shows a moderate improvement in oxidation and combustion performance among these materials. The results showed that a higher fluorine content in fluoropolymers will attribute to the better oxidation and combustion performance of boron powder. Additionally, fluoropolymer coating is a feasible way to improve the performance of boron powder. At present, the research and exploration of fluorine-containing polymer coating materials on boron powder are still insufficient.

### 3.3. Boron Powder Surface Modification with Metal and Metal Oxide

Metal is a common component in solid propellants, which can improve the detonation heat and density of propellants [98,99]. In addition, Al, Mg, Ti, Fe, and other metals can react with boron to generate metal borides and release heat at high temperatures. Metal borides have low ignition points, which is conducive to promoting the ignition and combustion of boron.

The advantage of metal oxides is that they are relatively stable and can be used as protective materials to prevent the oxidation of boron powder. Moreover, boron can react with metal oxides and generate metals and boron oxide. Subsequently, the generated metal can undergo the following two reactions: (1) as for the metal mentioned above, it reacts with boron to form metal borides to promote ignition; (2) it is further oxidized to metal oxides [100]. In this process, oxygen is transferred to the surface of boron particles faster through metal oxides, and metal oxides play a catalytic role in the whole oxygen transfer process [101,102].

#### 3.3.1. Boron Powder Coated with Metal

##### Boron Coated with Fe

**Figure 14:**
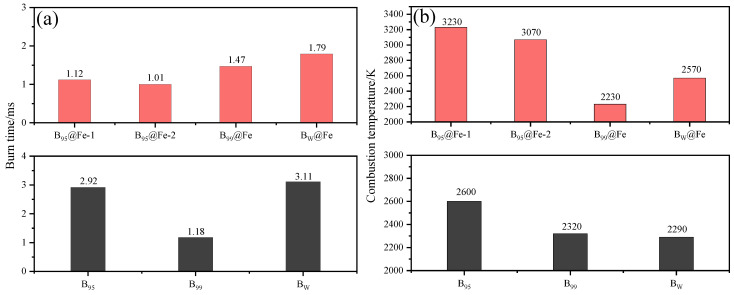
Coated and uncoated B_99_, B_95_, and B_w_ sample (**a**) burning times and (**b**) combustion temperatures (B_95_@Fe-1 and B_95_@Fe-2 were prepared by controlling the reaction temperature of Fe(CO)_5_ at 125 and 190 °C, respectively) [103,105].

Dreizin et al. have carried out a lot of research on Fe-coated boron powder [103,104,105]. Using the decomposition of Fe(CO)_5_ in octadecene (ODE) to form Fe nanoparticles [106], B@Fe was successfully prepared. As shown in Figure 14, B@Fe has a faster combustion rate and higher combustion temperature than B. Additionally, the higher-purity B_99_ (with purity greater than 99%) burned faster than the others before coating. However, the modification effect of B_95_ (with a purity higher than 95%) was the best after coating [105]. Compared with B_95_, the burning times of B_95_@Fe-1 and B_95_@Fe-2 decreased from 2.92 ms to 1.12 and 1.01 ms, respectively, and the burning temperatures increased from 2600 to 3230 and 3070 K, respectively. In addition, by washing B_95_ with acetonitrile, the authors also obtained B_w_ with the surface oxide layer removed. The improvement effect of B_99_@Fe and B_W_@Fe is not obvious, which is related to the deposition method of Fe coating. The decomposition of Fe(CO)_5_ occurs preferentially at the interface where the surface hydroxyl group exists, and B_95_ has a higher content of boron oxide that can react with water to generate a hydroxyl group. Therefore, the coating effect of B_95_ is much better than the others [103]. This also suggests that an improvement in boron powder purity or removal of the surface oxide layer will influence the Fe coating effect. This method provides a new idea for coating boron powder with metal.

##### Boron Coated with Mg

Magnesium powder is a common additive in propellant components, which has the advantages of low oxygen consumption, good ignition performance, and low molecular weight of combustion products. Qin [107] studied the ignition temperature of B@Mg, Mg, and aluminum alloy. In oxygen, the ignition temperature of B@Mg is only 195.92 °C, lower than that of Mg (326.58 °C) and aluminum alloy (270.49 °C). Coating magnesium on the surface of boron powder can effectively reduce the ignition point and promote the ignition and combustion of boron. In B@Mg, Mg can react with B_2_O_3_ to form B at a high temperature, which reduces the ignition time of boron particles and improves the combustion efficiency [108].

##### Boron Coated with Ti

Rosenband [109] theoretically studied the ignition and combustion behavior of B@Ti, which has a core–shell structure with 0.1–1 μm coating thickness. The results showed that uncoated boron particles need to be ignited at approximately 2000 K, whereas titanium-coated boron particles can be ignited at 1400 K. The proposed mechanism is as follows: (1) due to the reaction of titanium with boron and oxygen, the heating rate of particles increases; (2) TiO_2_ and TiB_2_ layers are destroyed by the thermal expansion mechanical stress generated by themselves. Therefore, the destroyed TiO_2_ and TiB_2_ layers cannot prevent boron in the inner layer from being oxidized.

#### 3.3.2. Boron Powder Coated with Metal Oxide

**Figure 15:**
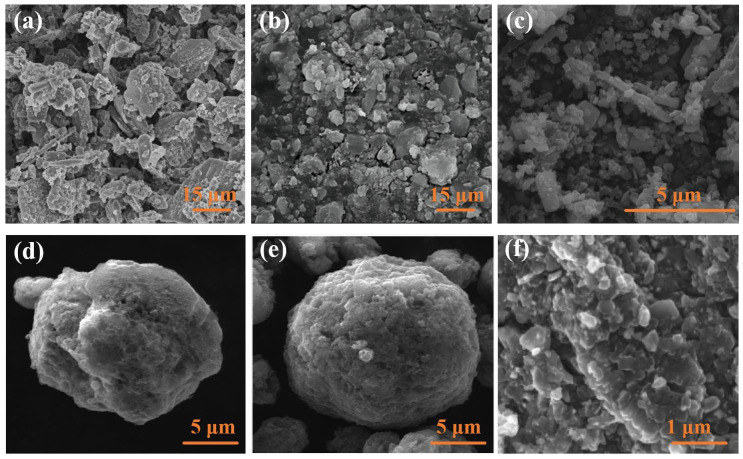
SEM images of (**a**) raw boron [110]; (**b**) B@NiO [111]; (**c**) B@CuO [112]; (**d**) B@SnO_2_ [113]; (**e**) B@TiO_2_ [113]; and (**f**) B@CeO_2_ [114].

The SEM images of different metal oxide-coated boron powders are shown in Figure 15; metal oxide nanoparticles coated on the surface of boron powder appeared in the form of microspheres. The irregularly shaped boron particles are covered by metal oxide nanoparticles, and the surface of the coated particles presents a pore structure.

##### Boron Coated with TiO_2_ or SnO_2_

**Figure 16:**
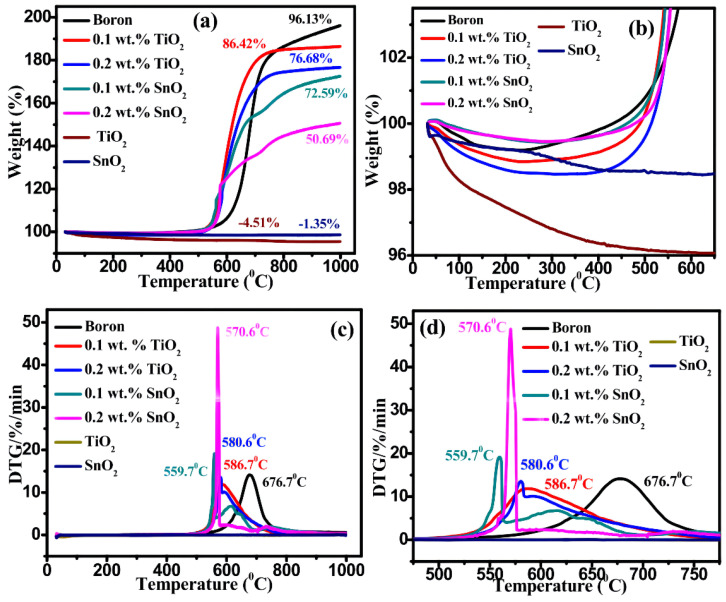
(**a**) TGA, (**b**) large view of TGA (25–675 °C), (**c**) DTG, and (**d**) large view of DTG (400–775 °C) of boron, TiO_2_, SnO_2_, and boron particles coated with 0.1 and 0.2 wt.% metal oxide (TiO_2_ or SnO_2_) in the presence of an air atmosphere [113].

Metal oxides are attractive candidates as combustion catalysts in propellants. Boron particles with metal oxide coatings have been synthesized using the spray-drying process [113], the wet-chemistry method [115,116], and chemical vapor deposition [117]. Lee [113] successfully prepared spherical boron nanoparticles coated with metal oxide (TiO_2_ or SnO_2_) by the spray-drying method. The diameter of the microspheres is in the range of 5–10 μm, showing a porous microsphere structure (Figure 15d,e). This structure has potential for applications in other fields, such as supercapacitors, batteries, and hydrogen storage materials. TGA-DTG analysis in Figure 16 indicates that metal oxide-coated boron particles have better oxidation resistance in air. In the DTG curves of Figure 16c,d, the exothermic peaks of B@SnO_2_ and B@TiO_2_ are advanced by up to 117 and 96.1 °C compared with raw boron. TiO_2_ and SnO_2_ particles do not show exothermic peaks in the DTG curves and neither of them shows a mass increase in the TGA curves; therefore, the exothermic peaks and mass increase can be attributed to the oxidation of boron. Similar results were observed in previous studies on B@Bi_2_O_3_ (104 °C) [110], B@NiO (30 °C) [111], B@CuO (116.86 °C) [112], B@SnO_2_ (160 °C) [115], and B@TiO_2_ (162 °C) [116].

Deshmukh [115,116] prepared B@SnO_2_ and B@TiO_2_ particles at room temperature with a simple wet-chemistry method. The thermal analysis results suggest the same conclusion as that obtained by Lee [113]. Furthermore, the ignition performance of the coated particles was studied. After the coating treatment, the ignition delay time of B@SnO_2_ and B@TiO_2_ was reduced to 227 μs and 190 μs, respectively, compared with 288 μs of the original boron.

**Figure 17:**
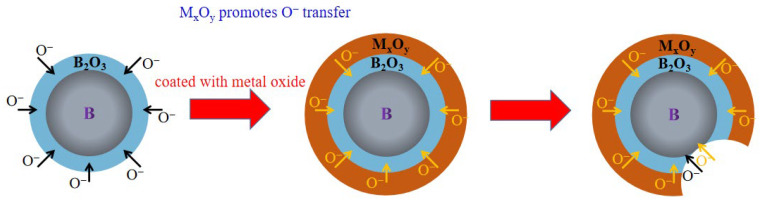
The reaction mechanism of B@M_x_O_y_.

The combustion mechanism of boron ignition promoted by metal oxide is shown in Figure 17. When boron is coated with the metal oxide, which are in close contact, boron can obtain O^−^ from metal oxides. The metal oxide promotes O^−^ to enter the inner shell and react with active B, thereby improving the combustion performance of boron powder. When the oxide layer is destroyed, metal oxides and boron can also form a thermite system to release a lot of energy.

**Figure 18:**
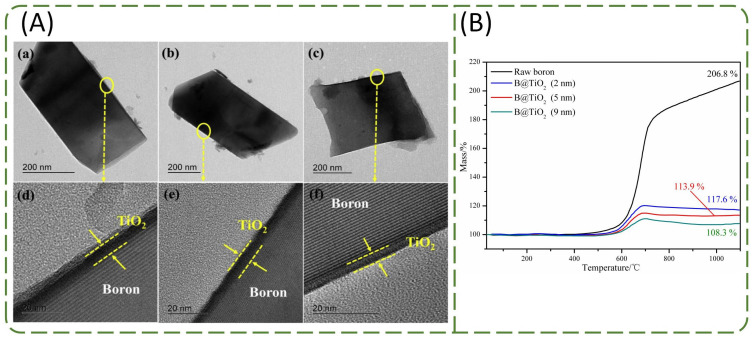
(**A**) TEM images of B@TiO_2_ in (**a**,**b**,**c**) low and (**d**,**e**,**f**) high resolution; (**B**) TGA curves of raw boron and B@TiO_2_ [117].

The chemical vapor deposition (CVD) method is a recently developed technology developed. In order to protect boron from oxidation, Sung [117] used the CVD method to prepare a uniform TiO_2_ cover layer on the surface of boron powder. It can be seen from Figure 18A that this layer is only a few nanometers thick and the TiO_2_ coating is uniform in thickness. TGA indicated that the mass change of boron particles with TiO_2_ coating is obviously smaller than that of bare boron particles (Figure 18B). When the thicknesses of the TiO_2_ coating layer were 2, 5, and 9 nm, the mass gains were 17.6, 13.9, and 8.3%, respectively. Therefore, the oxidation resistance of titanium dioxide-coated boron particles can be used to protect the surface of the boron oxide layer from further oxidation.

##### Boron Coating with CeO_2_

Devener [114] prepared B@CeO_2_ and studied the surface properties of B@CeO_2_ particles that were modified with oleic acid. B@CeO_2_ can form a stable colloidal solution in organic solvents and JP-5 fuels, which facilitates the dispersion of boron in liquid fuels. Additionally, the results indicate that CeO_2_-coated particles have a potential as fuel additives.

## 4. Comparative Analysis

### 4.1. Ignition and Combustion Performances

**Figure 19:**
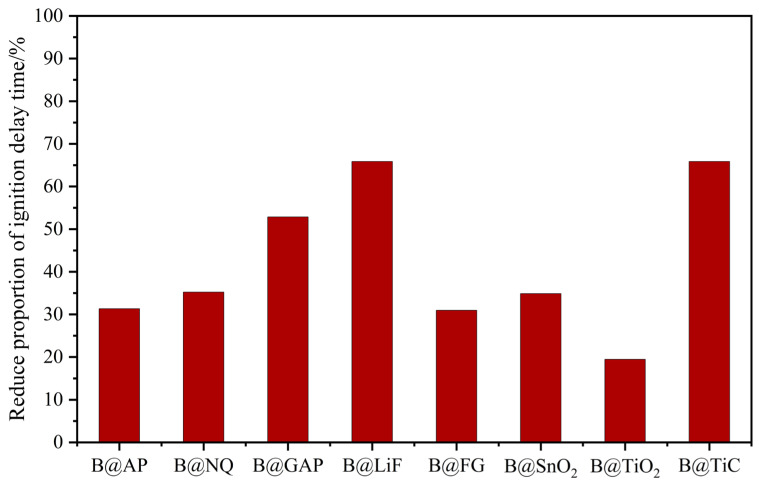
The reduction proportion of the ignition delay time [71,81,88,95,115,116,118].

Ignition delay time is one of the indices to characterize particle ignition performance. Due to the differences in ignition energy, the ignition delay time varies greatly in different studies. Therefore, the ignition delay time reductions in different materials were compared, as shown in Figure 19. In fact, all coating materials can improve the performance of boron. LiF and TiC exhibited the best ignition performance; the ignition delay time reduced by 66% [118]. The delay time reduction for LiF is attributed to the cleaning of the oxide layer on the surface of the boron powder by F^-^. On the other hand, the delay time reduction of TiC is attributed to the decomposition of carbides at high temperatures, which produces CO_2_ and destroys the oxide layer, thus promoting ignition.

**Figure 20:**
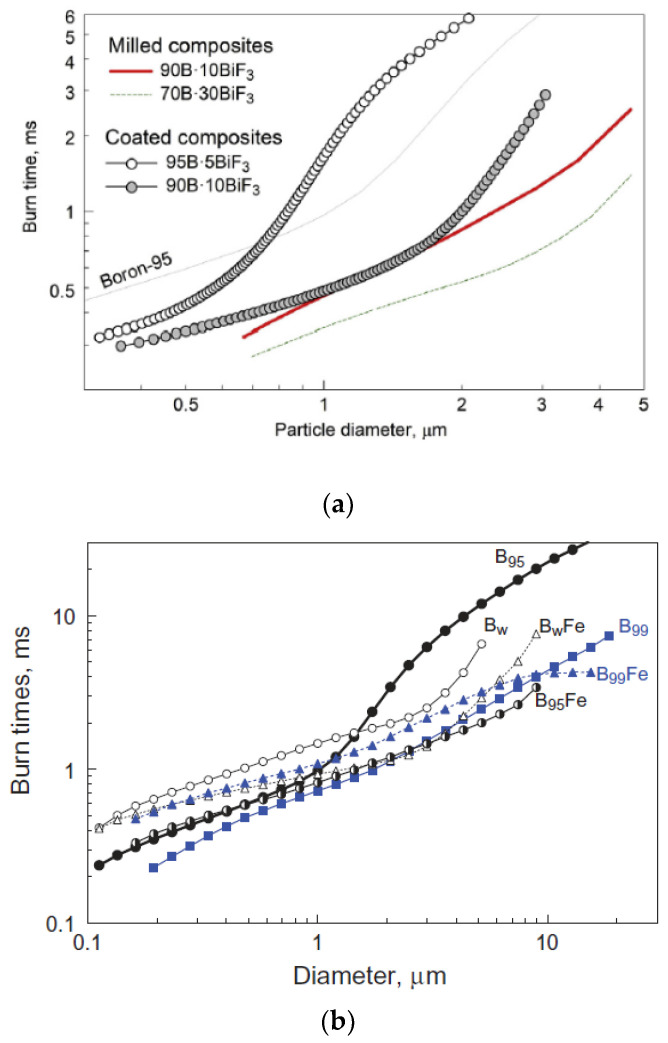
Correlation between the burning time and particle size of (**a**) B@BiF_3_ [92]; and (**b**) B@Fe [103].

Combustion temperature and burning time are important measurement indices of the sample reactivity. A higher combustion temperature and shorter burning time are usually expected. Table 3 shows that the combustion temperature is related to the type of coating agent. The combustion temperatures of B@AP and B@HMX increase, whereas the temperatures of B@LiP and B@KNO_3_ decrease, and the hygroscopicity of LiP and the low oxygen content of KNO_3_ may be the reasons for the decrease in combustion temperature. Fluoropolymer and metal exhibit clear improvements in the combustion temperature of boron powder. In particular, the combustion temperature of THV increased by 820 °C due to its high fluorine content. The burning time is typically a function of many parameters, such as particle size, oxide layer thickness, pressure, and gas composition. The burning time increases with the increase in boron particle size; however, the burning time of coated boron powder is shorter, as shown in Table 3 and Figure 20.

### 4.2. Thermal Reaction Performance

Table 4 summarizes the effects of different coating agents and preparation methods on the thermal reaction performance of boron powder.

Table 4 shows that the recrystallization method is usually employed for energized oxidant coatings of boron powder. In comparison, the initial oxidation temperature of boron was improved more significantly by coating with LiP and KNO_3_, and the temperature was reduced by more than 60 °C. The initial oxidation temperatures of HMX- and AP-coated boron powder were reduced by 12.1 and 15.7 °C, respectively. However, the heat release value and combustion temperature of boron coated with HMX and AP were 100–400 J·g^−1^ and 200–300 °C higher than those of boron coated with LiP and KNO_3_, respectively. In addition, the decomposition peak temperature of oxidants decreased in varying degrees except for HMX, indicating that the coating treatment can improve the reactivity of energetic oxidants [29,76]. When LiF was used as coating agent, the T_on_ (incipient oxidation temperature of boron) was reduced by 120 °C because the surface oxide layer of boron powder could be easily removed under the action of LiF. However, the T_on_ was only slightly improved by coating fluorine-containing polymers such as PVDF, Viton, and THV; the oxidation heat, reactivity, and combustion temperature of fluorine-containing polymers could be highly improved. With the increase in fluorine content (PVDF < Viton < THV), the improvement degree gradually increased. The combustion temperature of boron powder reached 2957 °C when Fe was used as a coating agent, and the ignition temperature of boron decreased by more than 40% when Mg was used as the coating agent. In addition, metal oxides played an important role in improving the reactivity of boron powder. The initial oxidation temperature of metal oxide (TiO_2_ and SnO_2_)-coated boron powder prepared by the chemical precipitation method and spray-drying method decreased significantly. However, the weight gains of metal oxide-coated boron powder prepared by the spray-drying method were significantly reduced in an oxidizing environment, which is more suitable for protecting boron. The weight gains of coated boron powder prepared by the chemical precipitation method were higher, which is suitable for preparing boron powder with high reactivity.

### 4.3. Boron-Based Propellants and Explosives

Detonation heat (Qv) is the heat released from the self-oxygen combustion of boron-based fuel-rich propellants, which reflects the potential energy content in the primary combustion process. The combustion heat (Hv) is the combustion heat released under the condition of sufficient oxygen, which reflects the sufficiency of propellant secondary combustion. Table 5 lists the detonation heat and combustion heat data of boron-based propellants presented in the literature.

Compared with uncoated boron powder, B@AP can improve the detonation heat of propellant, and the detonation heats of propellants coated with 10, 20, 30% AP increased by 2–4%, 5–6%, and 6%, respectively [29]. Moreover, the detonation heat of DNTF-based B@AP-containing explosive could reach 7696 kJ/kg, while that of the same formula containing uncoated boron explosive was 7208 kJ/kg [72]. In addition, the combustion heat of boron-based fuel-rich propellant increased by 1698 kJ/kg, and the detonation heat increased by 196 kJ/kg after LiF coating [91]. Cheng et al. [119] prepared B/NC/Fe composite particles, and the detonation heat and combustion heat of HTPB/AP-based propellant containing B/NC/Fe increased by 985 and 3470 kJ/kg, respectively. These experimental data show that coating treatment is a feasible way to improve the energy release efficiency of boron-based propellants or explosives.

## 5. Conclusions and Perspectives

As a kind of metal fuel, boron powder has a prominent advantage in calorific value and a broad prospect in explosives and propellants. This paper reviews and summarizes the ignition and combustion mechanism and surface modification methods for boron powder. The complex ignition and combustion mechanism of boron powder and inefficient energy release limit its application in propellants and explosives. Additionally, the following points remain to be further studied:

(1) Further research on the ignition and combustion behaviors of boron particles should been carried out, including the effects of the thermal mechanical properties, thickness, particle size, and crystal transformation of boron oxide shells on the ignition and combustion of boron particles. The effects of the coating layer, environmental atmosphere, and propellant additives on the behaviors of boron ignition and combustion should be determined, and the coating thickness and thickness control mechanism for fluoride, metal, and other coatings can be further explored through molecular dynamics simulations of the interactions between the coating and boron powder.
(2) The development of new package-covering materials with more comprehensive performance, and new package-covering methods with controllable package-covering technology are urgently needed. In view of the performance defects of single-coating materials, the combustion performance of binary component coatings, or even ternary component coating materials should be studied.
(3) To date, most of the research has been limited to basic studies on the ignition and combustion performance of boron powder coating modifications, which are limited to the laboratory. It is necessary to study the relationship between coating modification strategies and propellants. For example, the relationship between the performance parameters of propellants such as specific impulses, the critical pressure of propellant combustion chambers, pressure temperature coefficients, and the coating material of boron powder could be the focus of future research. Finally, more work is needed for the applications of coating boron powder at an industrial level.

## Figures and Tables

**Table 2:** The changes in viscosity with time in the mixing process of B and B@PBT with HTPB [80].

Viscosity/Pa·s	Stir Time/Min							
	20	40	60	80	100	120	140	160
HTPB + B	4000	9000	13,200	16,400	19,000	22,600	25,000	28,000
HTPB + B@PBT	26	835	530	800	960	1225	1364	1389

**Table 3:** Combustion temperature and burning time of coated boron powders.

Samples	Combustion Temperature/°C		Burning Time/ms	
	Before Coating	After Coating	Before Coating	After Coating
B@AP [76]	960.27	1116.24	–	–
B@LiP [76]	960.27	901.02	–	–
B@KNO_3_ [76]	960.27	826.87	–	–
B@HMX [76]	960.27	1163.92	–	–
B@GAP [81]	–	–	47.1	31.5
B@BiF_3_ [92]	–	–	3.4	1.09
B@PVDF [97]	1817	2077	–	–
B@Viton [97]	1817	2607	–	–
B@THV [97]	1817	2637	–	–
B@Fe [105]	2327	2797	2.92	1.01

**Table 4:** Properties of common materials coated with boron powder.

Samples	CC	FM	T_on_/°C	ΔT/°C	Oxidation Heat/J·g^−1^	Δm/%
B@AP [76]	10	recrystallization method	704.1	15.7	8966	35.37
B@LiP [76]	10	recrystallization method	640.4	79.4	8865	32.27
B@HMX [76]	10	recrystallization method	707.7	12.1	9110	34.27
B@KNO_3_ [76]	10	recrystallization method	659.6	60.2	8618	33.23
B@LiF [91]	10	neutral precipitation method	599	120	–	80.6
B@LiF [90]	10	neutral precipitation method	599	114	–	205.6
B@PVDF [97]	4	recrystallization method	747	7	7500	89.2
B@Viton [97]	4	recrystallization method	741	13	7900	91.2
B@THV [97]	4	recrystallization method	745	9	8200	92.9
B@TiO_2_ [116]		wet-chemistry method	665.4	135.7	–	94.2
B@TiO_2_ [113]	0.2	spray-drying method	580.6	96.1	–	76.7
B@SnO_2_ [113]	0.2	spray-drying method	570.6	106.1	–	50.7
B@SnO_2_ [115]		wet-chemistry method	400	50	–	99.2

Notes: CC: coating content, in wt.%; T_on_: incipient oxidation temperature of boron, in °C; Δm: total weight gain of boron powder oxidation, in %.

**Table 5:** Detonation heat (Qv) and combustion heat (Hv) of coated boron powder.

Samples	Qv/kJ·kg^−1^		Hv/kJ·kg^−1^	
	Before Coating	After Coating	Before Coating	After Coating
Prop. B@AP [29]	3974	4247	–	–
Prop. B@LiF [91]	4553	4749	26,017	27,715
Expl. B@AP [72]	7221	7695	–	–
Prop. B/Fe/NC [119]	6592	7577	10,380	13,850

## Data Availability

No new data were created or analyzed in this study. Data sharing is not applicable to this article.
